# Supplementary material for: Recent Advances in Biosensor Technologies for Point-of-Care Urinalysis
Source: Biosensors (Basel). 2022 Nov 15;12(11):1020. doi: 10.3390/bios12111020 (PMC9688579; doi:10.3390/bios12111020)
Supplement: Supplementary file 1 [file biosensors-12-01020-s001.zip › biosensors-1977318-supplementary.pdf]

biosensors

MDPI

Supporting Information

Recent Advances in Biosensor Technologies for Point-of-Care Urinalysis

Chuljin Hwang [1], Won-June Lee [2], Su Dong Kim [3], Sungjun Park [1,4,*] and Joo Hee Kim [5,*]

[1] Electrical and Computer Engineering, Ajou University, Suwon 16499, Republic of Korea
[2] Department of Chemistry, Purdue University, West Lafayette, IN 47907, USA
[3] Graduate School of Clinical Pharmacy and Pharmaceutics, Ajou University, Suwon 16499, Republic of Korea
[4] Leading Convergence of Healthcare and Medicine, Institute of Science & Technology (ALCHeMIST), Ajou University, Suwon 16499, Republic of Korea
[5] College of Pharmacy, Ajou University, Suwon 16499, Republic of Korea
* Correspondence: sj0223park@ajou.ac.kr (S.P.); elisekim@ajou.ac.kr (J.H.K.)

Table S1. Selected operators and search items and terms for the statistical publication analysis of "biosensors developed to identify urine-based biomarkers that are typically detected by dipstick tests."

Groups	Search Items and Terms
(A) Samples	"body (AND) fluids" OR "urine"
(B) Biomarkers	"hydrogen (AND) ion" OR "nitrites" OR "protein" OR "blood" OR "bilirubin" OR "glucose" OR "ketone" OR "leukocytes (AND) esterase" OR "sodium" OR "chloride" OR "iodide"
(C) Devices	"sensor" OR "biosensor" OR "detector"

Table S2. Summary of previously reported review papers of biosensors for several biomarkers

Biomarkers	Review papers
Glucose	Fenoy, G.E.; Marmisollé, W.A.; Knoll, W.; Azzaroni, O. Highly Sensitive Urine Glucose Detection with Graphene Field-Effect Transistors Functionalized with Electropolymerized Nanofilms. *Sens. Diagn*. **2022**, 10.1039.D1SD00007A, doi:10.1039/D1SD00007A.
	Huang, C.; Hao, Z.; Wang, Z.; Zhao, X.; Wang, H.; Li, F.; Liu, S.; Pan, Y. A Fully Integrated Graphene-Polymer Field-Effect Transistor Biosensing Device for on-Site Detection of Glucose in Human Urine. *Materials Today Chemistry* **2022**, 23, 100635, doi:10.1016/j.mtchem.2021.100635.
pH	Rabboh, F.M.; O'Neil, G.D. Voltammetric PH Measurements in Unadulterated Foodstuffs, Urine, and Serum with 3D-Printed Graphene/Poly(Lactic Acid) Electrodes. *Anal. Chem.* **2020**, 92, 14999–15006, doi:10.1021/acs.analchem.0c02902.
	Lee, S.; Park, S.; Kim, C.-H.; Yoon, M.-H. Approaching the Nernst Detection Limit in an Electrolyte-Gated Metal Oxide Transistor. *IEEE Electron Device Lett.* **2021**, 42, 50–53, doi:10.1109/LED.2020.3040149.
	Chae, M.-S.; Park, J.H.; Son, H.W.; Hwang, K.S.; Kim, T.G. IGZO-Based Electrolyte-Gated Field-Effect Transistor for in Situ Biological Sensing Platform. *Sensors and Actuators B: Chemical* **2018**, 262, 876–883, doi:10.1016/j.snb.2018.02.090.
Protein	Kim, B.; Kim, T.H. Determination of Human Serum Albumin Using a Single-Walled Carbon Nanotube-FET Modified with Bromocresol Green. *Microchim Acta* **2016**, 183, 1513–1518, doi:10.1007/s00604-016-1815-6.

	Zhang, G.; Yu, Y.; Guo, M.; Lin, B.; Zhang, L. A Sensitive Determination of Albumin in Urine by Molecularly Imprinted Electrochemical Biosensor Based on Dual-Signal Strategy. *Sensors and Actuators B: Chemical* **2019**, 288, 564–570, doi:10.1016/j.snb.2019.03.042.
	A. Karim, S.S.; Nadzirah, Sh.; Kazmi, J.; A. Rahim, R.; Dee, C.F.; Hamzah, A.A.; Mohamed, M.A. Zinc Oxide Nanorods-Based Immuno-Field-Effect Transistor for Human Serum Albumin Detection. *J. Mater. Sci.* **2021**, 56, 15344–15353, doi:10.1007/s10853-021-06288-0.
Ketone	Go, A.; Park, S.R.; Ku, Y.; Sun, M.; Yeon, S.; Lee, J.-K.; Lee, S.W.; Lee, M.-H. Highly Sensitive Electrochemical Sensor for Diagnosis of Diabetic Ketoacidosis (DKA) by Measuring Ketone Bodies in Urine. *Sensors* **2021**, 21, 4902, doi:10.3390/s21144902.
Blood	Anirudhan, T.S.; Alexander, S. A Potentiometric Sensor for the Trace Level Determination of Hemoglobin in Real Samples Using Multiwalled Carbon Nanotube Based Molecular Imprinted Polymer. *European Polymer Journal* **2017**, 97, 84–93, doi:10.1016/j.eurpolymj.2017.09.048.
Nitrite	Zhou, Y.; Ma, M.; He, H.; Cai, Z.; Gao, N.; He, C.; Chang, G.; Wang, X.; He, Y. Highly Sensitive Nitrite Sensor Based on AuNPs/RGO Nanocomposites Modified Graphene Electrochemical Transistors. *Biosensors and Bioelectronics* **2019**, 146, 111751, doi:10.1016/j.bios.2019.111751.
	Cardoso, R.M.; Silva, P.R.L.; Lima, A.P.; Rocha, D.P.; Oliveira, T.C.; do Prado, T.M.; Fava, E.L.; Fatibello-Filho, O.; Richter, E.M.; Muñoz, R.A.A. 3D-Printed Graphene/Polylactic Acid Electrode for Bioanalysis: Biosensing of Glucose and Simultaneous Determination of Uric Acid and Nitrite in Biological Fluids. *Sensors and Actuators B: Chemical* **2020**, 307, 127621, doi:10.1016/j.snb.2019.127621.
Bilirubin	Thangamuthu, M.; Gabriel, W.; Santschi, C.; Martin, O. Electrochemical Sensor for Bilirubin Detection Using Screen Printed Electrodes Functionalized with Carbon Nanotubes and Graphene. *Sensors* **2018**, 18, 800, doi:10.3390/s18030800.
	Rahman, M.M.; Ahmed, J.; Asiri, A.M. Selective Bilirubin Sensor Fabrication Based on Doped IAO Nanorods for Environmental Remediation. *New J. Chem.* **2019**, 43, 19298–19307, doi:10.1039/C9NJ05477D.
Leukocyte esterase	Ho, M.-L.; Liu, W.-F.; Tseng, H.-Y.; Yeh, Y.-T.; Tseng, W.-T.; Chou, Y.-Y.; Huang, X.-R.; Hsu, H.-C.; Ho, L.-I.; Pan, S.-W. Quantitative Determination of Leukocyte Esterase with a Paper-Based Device. *RSC Adv.* **2020**, 10, 27042–27049, doi:10.1039/D0RA03306E.
NMP-22	Wu, D.; Wang, Y.; Zhang, Y.; Ma, H.; Yan, T.; Du, B.; Wei, Q. Sensitive Electrochemical Immunosensor for Detection of Nuclear Matrix Protein-22 Based on NH2-SAPO-34 Supported Pd/Co Nanoparticles. *Sci Rep* **2016**, 6, 24551, doi:10.1038/srep24551.
	Li, Y.; Zeng, B.; Yang, Y.; Liang, H.; Yang, Y.; Yuan, Q. Design of High Stability Thin-Film Transistor Biosensor for the Diagnosis of Bladder Cancer. *Chinese Chemical Letters* 2020, 31, 1387–1391, doi:10.1016/j.cclet.2020.03.043.
	Yang, Y.; Wang, J.; Huang, W.; Wan, G.; Xia, M.; Chen, D.; Zhang, Y.; Wang, Y.; Guo, F.; Tan, J.; et al. Integrated Urinalysis Devices Based on Interface-Engineered Field-Effect Transistor Biosensors Incorporated With Electronic Circuits. *Advanced Materials* **2022**, 34, 2203224, doi:10.1002/adma.202203224.
PSA	Pal, M.; Khan, R. Graphene Oxide Layer Decorated Gold Nanoparticles Based Immunosensor for the Detection of Prostate Cancer Risk Factor. *Analytical Biochemistry* **2017**, 536, 51–58, doi:10.1016/j.ab.2017.08.001.
miR-107	Koo, K.M.; Carrascosa, L.G.; Shiddiky, M.J.A.; Trau, M. Poly(A) Extensions of MiRNAs for Amplification-Free Electrochemical Detection on Screen-Printed Gold Electrodes. *Anal. Chem.* **2016**, 88, 2000–2005, doi:10.1021/acs.analchem.5b04795.

Sodium ions	Oh, H.G.; Jeon, D.C.; Gianti, M.S.; Cho, H.S.; Jo, D.A.; Indriatmoko, M.N.; Jang, B.K.; Lim, J.M.; Cho, S.; Song, K.S. Two–Dimensional Disposable Graphene Sensor to Detect Na+ Ions. *Nanomaterials* **2021**, 11, 787, doi:10.3390/nano11030787.
Potassium ions	Iannazzo, D.; Espro, C.; Ferlazzo, A.; Celesti, C.; Branca, C.; Neri, G. Electrochemical and Fluorescent Properties of Crown Ether Functionalized Graphene Quantum Dots for Potassium and Sodium Ions Detection. *Nanomaterials* **2021**, *11*, 2897, doi:10.3390/nano11112897.
Chloride ions	Cunha-Silva, H.; Julia Arcos-Martinez, M. Development of a Selective Chloride Sensing Platform Using a Screen-Printed Platinum Electrode. *Talanta* **2019**, *195*, 771–777, doi:10.1016/j.talanta.2018.12.008.
Iodide ions	Khunseeraksa, V.; Kongkaew, S.; Thavarungkul, P.; Kanatharana, P.; Limbut, W. Electrochemical Sensor for the Quantification of Iodide in Urine of Pregnant Women. *Microchim Acta* **2020**, *187*, 591, doi:10.1007/s00604-020-04488-0.
	Hwang, C.; Kwak, T.; Kim, C.-H.; Kim, J.H.; Park, S. Quantitative and Rapid Detection of Iodide Ion via Electrolyte-Gated IGZO Thin-Film Transistors. *Sensors and Actuators B: Chemical* **2022**, *353*, 131144, doi:10.1016/j.snb.2021.131144.

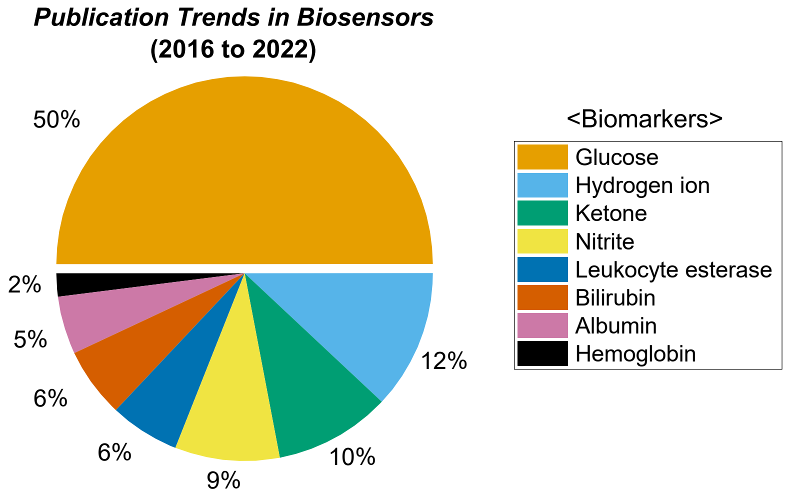

Figure S1**.** Publication trends in the biosensor research field based on the PubMed, Scopus, IEEE Xplore, Google Scholar, and Embase electronic databases from 2016 to 2022 for detecting urine-based biomarkers that are traditionally detected by dipstick tests. (Percentage of the number of the biosensors for specific biomarkers to the number of urine-based biosensors publications)
